# Aerosolized polymyxins for ventilator-associated pneumonia caused by extensive drug resistant Gram-negative bacteria: class, dose and manner should remain the trifecta

**DOI:** 10.1186/s13613-022-01068-8

**Published:** 2022-10-17

**Authors:** Jean-Jacques Rouby, Yinggang Zhu, Antoni Torres, Jordi Rello, Antoine Monsel

**Affiliations:** 1 Multidisciplinary Intensive Care Unit, Department of Anesthesiology and Critical Care, La Pitié-Salpêtrière Hospital, Assistance-Publique Hôpitaux de Paris, Sorbonne University of Paris, Paris, France; 2 Department of Pulmonary and Critical Care Medicine, Hua-Dong Hospital, Fudan University, Shanghai, China; 3 Department of Pneumology, SGR 911- Ciber de Enfermedades Respiratorias (Ciberes), Institut Clinic del Tórax, Hospital Clinic of Barcelona - Institut d’Investigacions Biomèdiques August Pi I Sunyer (IDIBAPS), University of Barcelona, Barcelona, Spain; 4 Centro de Investigación Biomédica en Red (CIBERES), Instituto de Salud Carlos III, Madrid, Spain; 5 Clinical Research & Innovation in Pneumonia & Sepsis, Vall d’Hebron Institute of Research (VHIR), Barcelona, Spain; 6 Clinical Research, CHU Nîmes, Université Montpellier-Nîmes, Nîmes, France; 7 Unité mixte de recherche (UMR)-S 959, Immunology-Immunopathology-Immunotherapy (I3), Institut National de La Santé et de La Recherche Médicale (INSERM), Paris, France; 8 Biotherapy (CIC-BTi) and Inflammation-Immunopathology-Biotherapy Department (DHU i2B), Hôpital Pitié-Salpêtrière, Assistance Publique-Hôpitaux de Paris, Paris, France

Improving the management of ventilator-associated pneumonia (VAP) represents an unmet clinical need, particularly when caused by carbapenem-resistant organisms, with need to optimize therapy to reduce adverse events, intubation period and length of ICU stay. Aerosolized antibiotics have been postulated as an opportunity to improve outcome, based on the large experience in chronic *Pseudomonas aeruginosa* bronchial infection in cystic fibrosis bronchiectasis. In contrast to non-ventilated patients, or with aerosolized steroids or brochodilators, aerosolized antibiotics among ventilated patients require a specific administration protocol [1] regarding devices and ventilator settings with need to minimize ventilator asynchronies for good patients’ tolerance. Interestingly, meta-analysis of randomized clinical trials [2], documented that replacement of the intravenous route by nebulization among ventilated patients reduce the risk of acute kidney injury; as a consequence, the European Society of Clinical Microbiology and Infectious Diseases endorsed in a position paper recommend this topic as an area of priority research [3].

In this issue of *Annals of Intensive Care*, Liu and co-workers report a Chinese retrospective multicenter observational matched (1:2) cohort study performed in patients with VAP caused by Extensive Drug Resistant (XDR) Gram-negative bacilli *(47.7% Klebsiella pneumoniae, and 40.2% Acinetobacter baumannii)* and treated either by a low intravenous (single-daily dose) of polymyxin B (PMB) or in combination with 1.8 mg/kg inhaled PMB [4]. Median maintenance IV doses (1 mg/kg/12 h) were 2/3 lower than the maintenance dose of 1.5 mg/kg/12 h recommended by the 2019 International Consensus Guidelines for the Optimal Use of the Polymyxins [5]. After matching on age, gender, septic shock, and Apache II score, the rates of clinical cure and bacterial eradication was less than 50% and did not show any significant difference between the two groups. Safety, VAP-related (27.3% vs. 34.1%) and global mortality were similar in both groups. The present study is in line with three randomized control trials reporting the lack of benefit to combine intravenous and nebulized antibiotics [6–8]. However, it shares one of the major methodological deficiencies of these studies: the use of too small nebulized doses that may explain therapeutic failure [9–11].

Authors suggested more “favorable clinical outcome” among patients treated with adjuvant inhaled PMB, without repercussions on earlier extubation rate or length of stay. However, survivors had a lower SOFA score, suggesting that other factors than antibiotic treatment may have accounted for the suggested benefit.

As summarized in the figure, using PMB rather than polymyxin E (PME) has several advantages: 1—PMB is a mixture of 4 polymyxins (PMB1–4), that are concentration-dependent antibiotics directly active against GNB. PME is a prodrug (colistimethate sodium) requiring in vivo hydrolysis to release colistin A and B, the active antibiotics against Gram-negative bacteria; 2—IV PMB penetrates in the infected lung parenchyma [12], although diffusion of IV colistimethate sodium in the epithelial lining fluid after IV administration is almost zero [13]. Conversely, nebulized PMB rapidly diffuses in the systemic compartment (Fig. 1B), whereas nebulized colistimethate sodium and resulting colistin have a very weak and slow diffusion in the systemic compartment (Fig. 1C); 3—PMB is less nephrotoxic than colistimethate sodium although exact mechanisms of renal toxicity are not fully understood [5]; 4—PMB has better PK/PD characteristics than PME that allows a rapid and reliable attainment of desired lung parenchymal and plasma concentrations in critically ill patients.

**Fig. 1:**
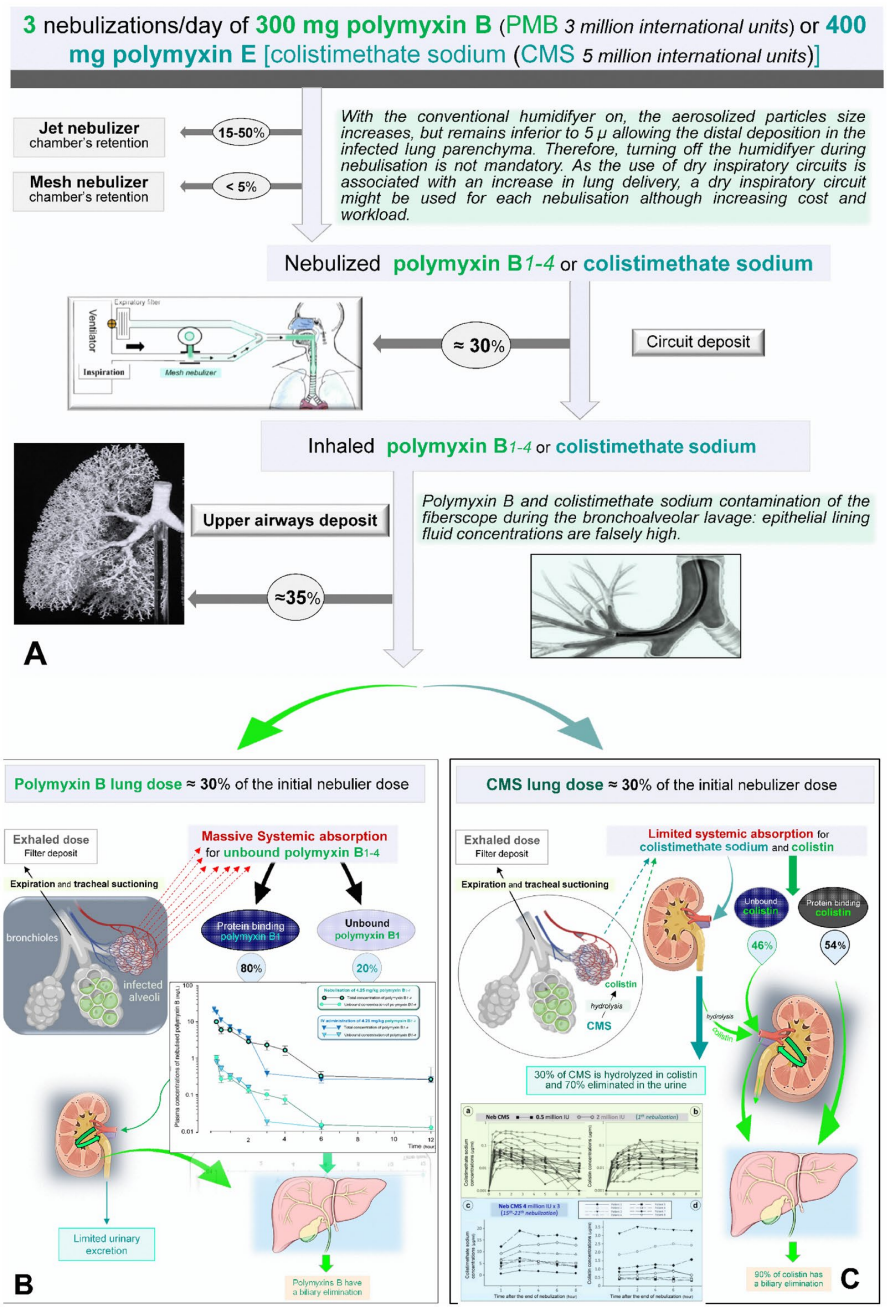
Nebulization of polymyxin B and E (colistimethate sodium) for treating ventilator-associated pneumonia caused by extensive drug resistant Gram-negative bacteria **A** Optimization of nebulization and recommended doses; **B** PK/PD after nebulization of high-dose polymyxin B. Very high doses are attained in the infected lung parenchyma and there is a massive systemic absorption. Because 80% of plasma polymyxin B binds to the proteins, plasma unbound polymyxin B Is not different after intravenous administration or nebulization of 4.25 mg/kg (square shows plasma concentrations according to time). Unbound polymyxin B is filtered and massively reabsorbed by the kidney so that hepatic elimination is predominant; **C** PK/PD after nebulization of high dose of colistimethate sodium. Very high doses are attained in the infected lung parenchyma and there is a low systemic absorption (yellow square shows individual plasma concentrations according to time after the first nebulization (**a** and **b**) of 0.5 and 2 million International Units 3/24H and blue square shows individual plasma concentrations after multiple nebulizations (**c** and **d**) of 4 million International Units 3/24H). Fifty five % of plasma colistin binds to the proteins and is eliminated by the liver. Thirty % of colistimethate sodium is hydrolyzed into colistin in the kidney and eliminated by the liver. PK/PD data are reproduced from *reference 14 with permission of the publisher*

PK/PD following IV administration and nebulization of PMB have been described in a mouse lung *Pseudomonas aeruginosa* infection model [14]. PMB has three original PK/PD characteristics: 1—it diffuses through the inflamed blood–air barrier (from the alveolar to the systemic compartment and from the systemic to the interstitial compartment); 2—it binds to proteins in a high proportion (80%); 3—PMB1–4 are concentration-dependent antibiotics which produce a potent bactericidal effect in the infected lung parenchyma. Combining nebulization and IV administration of colistimethate sodium has a weak rationale: PME poorly diffuses through the inflamed alveolar–capillary barrier and is nephrotoxic after IV administration. Apart from the minority case of bacteremic VAP, combining both routes of administration results in maintaining the risk of nephrotoxicity without increasing concentrations at the site of infection. This treatment schedule does not apply to PMB: nebulized and IV PMB massively diffuses through the inflamed alveolar–capillary barrier and the binding to plasma proteins protects against kidney exposure, reducing the risk of renal toxicity. Combining both routes of administration does not increase the risk of renal toxicity while increasing concentrations at the site of infection. In Liu’s study, combining both routes of administration may have accounted for the final more favorable outcome by interrupting bacterial penetration within the tracheobronchial tree [4].

Another key issue to achieve therapeutic efficiency of aerosolized PMB is the administration of adequate inhaled dose. Although there are no recommendations from academic societies, appropriate nebulized PMB dose might be inferred from PK/PD data [14] and the understanding of conditions required to penetrate into the infected lung parenchyma [15]. As PMB are concentration-dependent antibiotics, epithelial lining fluid concentrations should be tenfold the minimal inhibitory concentrations of the causative Gram-negative microorganisms. PK/PD studies indicate that 4.12 mg/kg/8 h is the dose to be inserted in the nebulizer’s chamber to achieve epithelial lining fluid concentrations at 20 µ/ml 8 h after the nebulization [14]. Therefore, the optimal nebulized dose should be 300 mg/8 h in a 70 kg critically ill patient. Each vial of PMB containing dry powder 50 mg (500,000 International Units) is dissolved in 2 mL of 0.9% sodium chloride to achieve a concentration of 250,000 International Units/ml. The 300 mg nebulization dose (6 vials reconstituted in 12 ml of 0.9% Sodium Chloride) is delivered by the mesh nebulizer in 30–45 min. In Liu’s study, the nebulized dose was low (1.82 mg/kg/12 h) as well as the intravenous dose (2 mg/kg), explaining why the combination of intravenous and aerosolized PMB was unable to treat efficiently VAP caused by XDR gram negative bacteria [4]. In China, the cost of PMB is not covered by all the personal medical insurances, and it would be a huge financial burden for most people, which may have a potential influence on the physicians' decision concerning the administered dose.

To provide high lung deposition in the infected lung parenchyma, mesh nebulizers should be preferred to jet nebulizers and the humidification system may be turned off during the nebulization phase (Fig. 1A), ventilator settings should be optimized during the nebulization phase to reduce extrapulmonary deposition: controlled ventilation with 40 l/min constant inspiratory flow (avoid pressure support and spontaneous breathing), respiratory frequency 12–15/min, I/E = 1:2, inspiratory pause 20%, positive-end expiratory pressure 5–10 cmH_2_O and asynchronies between the patient and the ventilator should be avoided by administering a short propofol sedation [15]. In Liu’s study, optimization of nebulization was achieved and may have accounted for the survival benefit [4]. Finally, the failure of a combination of intravenous and nebulized PMB to treat efficiently VAP caused by XDR Gram-negative bacteria, was likely due to the administration of low doses rather than to the failure of the antibiotic treatment itself.

Further efforts should be done to avoid the current heterogeneity in devices and clinical practices [16], needing to improve the strategy of administration. A priority should be to standardize the administration of aerosol antibiotics among ventilated patients with respiratory infections, with evidence-based clinical practices guidelines with multidisciplinary participation of physicians, nurses and respiratory therapists. Both MRSA and carbapenem-resistant Gram-negative bacilli should be the targets. A new paradigm is needed in the design of clinical research focusing on randomized clinical trials with superiority outcomes (rather than non-inferiority) as done in the oncologic research. Whether is the optimal therapy to treat VAP caused by XDR Gram-negative bacteria need to be elucidated with meaningful clinical outcomes as end-points. As far as polymyxins are concerned, an important limitation should be considered: the availability of PMB and PME worldwide [17–19]. Both polymyxins are available in North and South America and most countries of Southeast Asia and Oceania. Polymyxin E only is available in most European countries, Australia, Kenya, South Africa, Saudi Arabia, Cambodia and Vietnam. Polymyxins are not available in most African countries, Guatemala, Bolivia, Venezuela, Laos, Indonesia, Portugal, Norway, Japan and Russia. In countries where PMB is not available, the only option is the nebulization of PME. In countries where both polymyxins are available, nebulized PMB should be preferred to nebulized colistimethate sodium and three arms should be compared: IV PMB, aerosolized PMB and a combination of both. There is a strong rationale for delivering neb PMB as a sole treatment of VAP: in animals with inoculation pneumonia, it has been shown that nebulized amikacin and PME penetrate in consolidated lung areas and that tissue concentrations in non-aerated lung regions are largely above minimal inhibitory concentrations of susceptible Gram-negative bacteria (13, 20–23). In addition, comparison of aerosolized high-dose PME versus new intravenous combinations of latest-generation antibiotics (e.g., cephalosporins associated with beta-lactamase inhibitors) with improved activity against carbapenem-resistant bacteria, with a superiority design, are also required. A new paradigm of research, mimicking trials in cancer for severe infections is warranted.

## Data Availability

Not applicable.
